# Ferroelectric domain-wall logic units

**DOI:** 10.1038/s41467-022-30983-4

**Published:** 2022-06-06

**Authors:** Jing Wang, Jing Ma, Houbing Huang, Ji Ma, Hasnain Mehdi Jafri, Yuanyuan Fan, Huayu Yang, Yue Wang, Mingfeng Chen, Di Liu, Jinxing Zhang, Yuan-Hua Lin, Long-Qing Chen, Di Yi, Ce-Wen Nan

**Affiliations:** 1 Advanced Research Institute of Multidisciplinary Science, and School of Materials Science and Engineering, Beijing Institute of Technology, Beijing, 100081 China; 2 State Key Laboratory of New Ceramics and Fine Processing, School of Materials Science and Engineering, Tsinghua University, Beijing, 100084 China; 3 School of Materials Science and Engineering, Kunming University of Science and Technology, Kunming, Yunnan 650093 China; 4 Department of Physics, Beijing Normal University, Beijing, 100875 China; 5 Department of Materials Science and Engineering, Pennsylvania State University, University Park, PA 16802 USA

**Keywords:** Electronic devices, Ferroelectrics and multiferroics

## Abstract

The electronic conductivities of ferroelectric domain walls have been extensively explored over the past decade for potential nanoelectronic applications. However, the realization of logic devices based on ferroelectric domain walls requires reliable and flexible control of the domain-wall configuration and conduction path. Here, we demonstrate electric-field-controlled stable and repeatable on-and-off switching of conductive domain walls within topologically confined vertex domains naturally formed in self-assembled ferroelectric nano-islands. Using a combination of piezoresponse force microscopy, conductive atomic force microscopy, and phase-field simulations, we show that on-off switching is accomplished through reversible transformations between charged and neutral domain walls via electric-field-controlled domain-wall reconfiguration. By analogy to logic processing, we propose programmable logic gates (such as NOT, OR, AND and their derivatives) and logic circuits (such as fan-out) based on reconfigurable conductive domain walls. Our work might provide a potentially viable platform for programmable all-electric logic based on a ferroelectric domain-wall network with low energy consumption.

## Abstract (web summary)

Ferroelectric domain walls have been extensively explored for nanoelectronics, but the logic devices are still challenging. Here, the authors propose programmable logic gates and circuits based on electric-field controllable conductive domain walls.

## Introduction

Domain walls (DWs) in ferroic materials have attracted intensive interest over the past decades due to their physical phenomena and potential for applications in nanoelectronics^1,2^ and spintronics^3–5^. Of particular interest are the designs of racetrack memory^6,7^ and DW logic^8,9^ based on moving magnetic DWs driven by a current or a magnetic field. In these cases, data storage or logic operation functions could be realized by controlling DW injection, motion, and annihilation along magnetic nanowires rather than switching magnetic domains as is traditional. Contrary to the large wall width and high energy cost in current-driven moving magnetic DWs, ferroelectric DWs possess a much smaller wall width (less than a few nanometers), and their electric-field-driven features and electric conduction are conducive to high-density integration and low-power regulation of nanoelectronic devices^1,10–25^. Recently, by controlling the electric conduction of ferroelectric DWs with a low voltage, low or high resistance states could be created to realize 0 or 1 data bits for memory applications^18–23,25^. For example, a prototype nonvolatile DW memory unit was demonstrated based on reconfigurable charged DWs (CDWs)^19–21,23^, and the readout current of DWs hinged on the conformational changes of the DWs driven by an electric field between two in-plane terminal electrodes. More intriguingly, ferroelectric DW memory was reported based on a high-density CDW array with a controllable readout current^18^.

Thus, CDWs can be used as dynamic functional entities, and their large local electric conduction also offers intriguing possibilities for potential nanometer-sized logic applications. Analogous to magnetic racetrack memory or DW logic in nanomagnetic, a ferroelectric DW diode and a field-effect transistor have recently been proposed. The former one is based on the unidirectional motion of all DWs in a terraced KTiOPO_4_ crystal lamella;^26^ the latter one is designed through erasing and rewriting of CDWs between the drain, gate, and source electrodes on a LiNbO_3_ single crystal^25^. However, unlike magnetic DW logic in nanowires, the realization of ferroelectric DW logic is still a great challenge. The key issue is the implementation and integration of the CDW network with one CDW independently controlled without disturbing the other CDWs. Here, we demonstrate that CDWs within topologically confined vertex domains naturally formed in self-assembled BiFeO_3_ nano-islands^18,27,28^ can provide stable networks of local conductive paths for ferroelectric DW logic. Such a ferroelectric CDW network controllable by an electric field with nonvolatile conductance constitutes the building blocks for the proposed reconfigurable DW logic gates and circuits, indicating potential applications for all-electric memory-in-logic devices. By comparing with emerging magnetic DW devices^6–9^, the ferroelectric DW logic units exhibit higher integration density due to a much smaller DW width, fast operating speed, and lower energy consumption due to the electric-field-driven features.

## Results

### Electric control of DW morphology and logic operation analogue

**Fig. 1:**
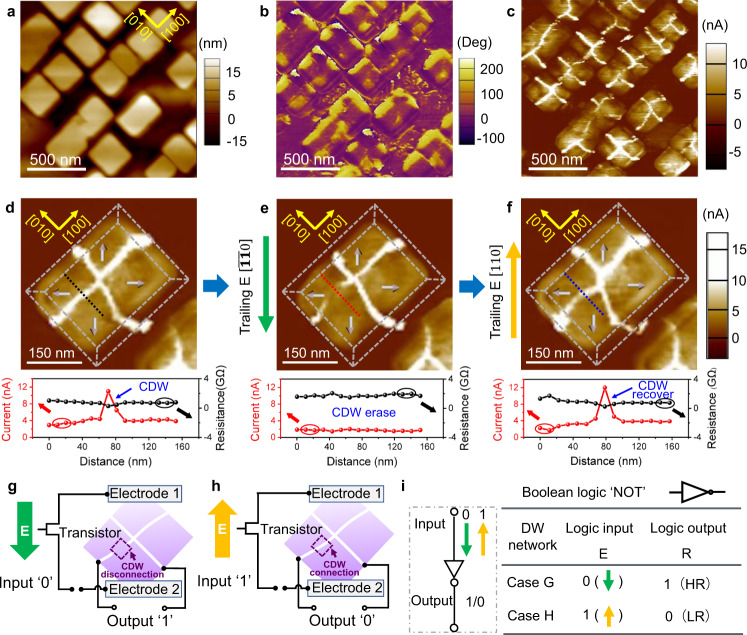
Electric field control of the on-and-off switching of a CDW and a NOT gate. **a** Morphology, **b** in-plane PFM phase, and (**c**) c-AFM image of vertex domains confined in a rectangular BiFeO_3_ nano-island array. **d** Initial cross-shaped CDW network with center-divergent (marked by three-dimensional arrows) vertex domains confined in a rectangular BiFeO_3_ nano-island. **e** Disconnection of a CDW branch by a [$\bar{1}\bar{1}0$]-oriented (marked by the green arrow) trailing field. **f** Reconnection of this disrupted CDW branch by a [$110$]-oriented (marked by yellow arrow) trailing field. The curves below panels (**d**–**f**) are the corresponding current profiles along the dashed lines in (**d**–**f**). **g**, **h** Schematic of two logic operations of the NOT gate based on the experimental observations in (**e**, **f**), where [$\bar{1}\bar{1}0$]- and [$110$]-oriented electric fields are defined as logic inputs ‘0’ and ‘1’, and the corresponding high and low resistance (HR and LR) states for the disconnected and connected CDW are defined as logic outputs ‘1’ and ‘0’, respectively. **i** Corresponding symbol (left) and truth table (right) for the NOT logic gate.

CDWs can naturally form in BiFeO_3_ nano-islands^18,27–30^. By controlling the growth conditions (see “Methods”), we fabricated a BiFeO_3_ nano-island array with a size of 200–350 nm and an aspect ratio of 1.0–1.5, as shown in Fig. 1a and Fig. S1. Piezoresponse force microscopy (PFM) and conductive-atomic force microscopy (c-AFM) images (Fig. 1b, c and Fig. S2) show cross-shaped CDWs of vertex domains confined in each nano-island. The ferroelectric hysteresis loop (Fig. S3) and the corresponding PFM and c-AFM images (Fig. S4) indicate the reversible control of the quad-domain between center-convergent (Fig. S4a,b) and center-divergent (Fig. S4d,e) polarization states, which accompanies with the head-to-head CDWs with a low conductance state (~pA, Fig. S4c) and a tail-to-tail CWDs with a high conductance state (~nA, Fig. S4f)^18^. For the head-to-head CDWs in BiFeO_3_ nano-islands, the screening charges arise from the intrinsic electronic carriers (i.e., electrons liberated from oxygen vacancies) from n-type BiFeO_3_ nano-islands^21,31^. While for the tail-to-tail CDWs, the screening charges arise from the p-type LSMO electrode when the quad-domain switched from a downward center-convergent pattern to an upward center-divergent pattern under the applied electric field, where a large number of holes are delivered to the tail-to-tail CDW region to compensate the negative bound charges^18^.

To design ferroelectric DW logic based on CDWs, we first explore the dynamic behavior of the cross-shaped CDWs with a high conductance state under a probe-based trailing electric field^13,32,33^. The details of the control process can be seen in Supplementary Fig. 5. Intriguingly, a targeted CDW of the off-center vertex domains confined in a rectangular nano-island with an aspect ratio deviating from 1 can be independently disconnected and connected again by a trailing electric field without affecting the other CDWs, as shown in Fig. 1d–f. For the initial state in Fig. 1d, the conductive paths of the four CDW branches of the off-center vertex are connected. When an electric field of −3 V is applied on the nanoscale probe (Fig. S5a) with an in-plane downward slow-scanning direction (Fig. S5b), a trailing field along with the [$\bar{1}\bar{1}0$] direction is generated during probe scanning, and the targeted CDW branch is disconnected, as shown in Fig. 1e. This disconnected CDW can be reconnected by a trailing field along the [$110$] direction (Fig. S5c), as shown in Fig. 1f. The local current and the corresponding resistance state for the connected and disconnected CDW are recorded as shown in the lower panel of Fig. 1d–f. The reproducibility of the connection and disconnection of the CDW has been further confirmed by c-AFM measurements (Fig. S6), and the corresponding low and high resistance state is also recorded as shown in Fig. S7. According to our previous results on the reversible control of the CDW resistance states on the vertex domains of nano-islands^18^, the repeatable on-and-off switching for CDW network could be no less than 10^2^ cycles. Moreover, time- and temperature-dependent c-AFM measurements, as shown in Figs. S8 and S9, respectively, also illustrate the stability of the modulated CDW network. Such robust control characteristics of the cross-shaped CDWs of the off-center vertex confined in the rectangular nano-island allow reversible control of the ‘on’ and ‘off’ states of one CDW without interfering with the other CDWs. Notably, the CDWs in the square nano-island with an aspect ratio of ~1.0 show negligible variation, e.g., the four CDWs remain after applying the in-plane trailing field as shown in Fig. S10. This stability may arise from the topological protection of the square nano-island^18,30,34^.

Taking advantage of the reversible modulation of the local conductance states of the CDWs by a trailing field along with the [$\bar{1}\bar{1}0$] or [$110$] direction, we propose ferroelectric DW logic, as shown in Fig. 1g–i. The [$\bar{1}\bar{1}0$]- and [$110$]-oriented trailing fields are used to represent the input Boolean logic values ‘0’ and ‘1’, respectively, and the low and high conductance states between the two selected terminals of the cross-shaped CDWs represent the output Boolean logic values ‘1’ and ‘0’, respectively. Here, the transistors are required to control which unit works and which does not, as shown in Fig. 1g, h. Thus, the switching between disconnected (Fig. 1e and g) and connected (Fig. 1f and h) CDWs by an electric field along with the [$\bar{1}\bar{1}0$]- and [$110$]-orientations, respectively, is analogous to a ‘NOT’ gate, as illustrated by the schematic and truth table in Fig. 1i.

### Mechanism of connection and disconnection of ferroelectric DW

**Fig. 2:**
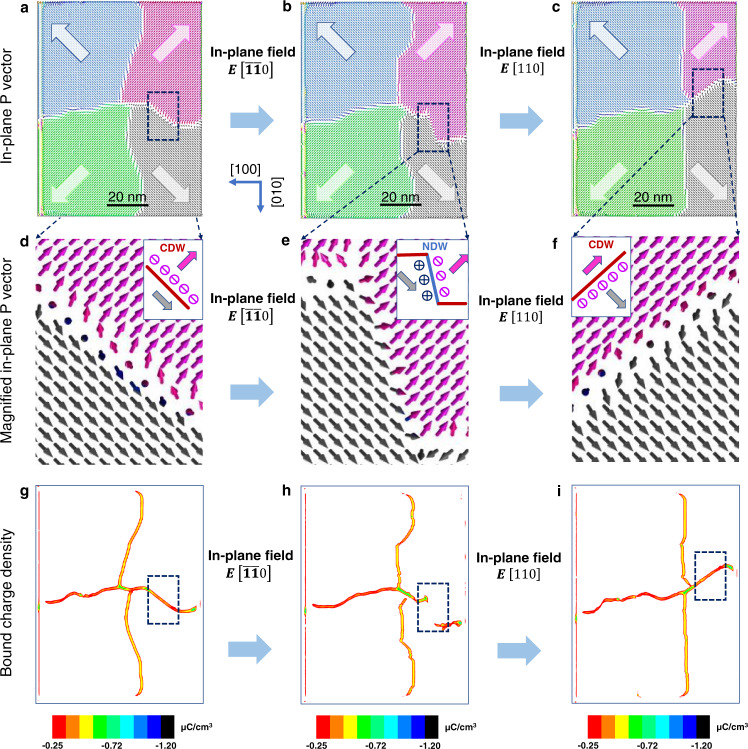
Mechanism of the tunability of CDWs by an electric field based on phase-field simulations. **a** Polar vector map for a 178 × 138 nm^2^ nano-island with cross-shaped CDWs. **b**, **c** Polar vector maps for the rectangular nano-island after a [$\bar{1}\bar{1}0$]- or [$110$]-oriented electric field is applied in the area enclosed by the blue dotted box in (**a**). **d**–**f** Magnified polar vector maps for the area highlighted by the blue dotted boxes in (**a**, **b**, **c**), which show that the local DW is reversibly modulated between a CDW and a neutral DW by the local electric field. **g**–**i** Corresponding charge density maps for (**a**, **b**, **c**). The connected CDWs in (**g**) and (**i**) and disconnected CDWs in (**h**) indicate a high conductance for the CDW and a low conductance for the neutral DW, respectively.

To understand the on-and-off switching of the CDW, we performed phase-field simulations to investigate the evolution of the CDW configuration in a rectangular nano-island under an in-plane electric field (see Fig. 2a–c and Supplementary Movies 1, 2). Considering metals may be used as contacts in each of the proposed logic units, different screening charge densities were added to simulate the domain structures in the nano-island, as shown in Fig. S11. As seen, the four-fold quad-domains remain either for the partially compensated state or for the fully compensated state. In Fig. 2, 156 μC/cm^2^ charges were added to 45° edges of the nano-island, which means the in-plane polarization induced bound charges have been fully compensated^35^. The initial vertex state of the nano-island (178 × 138 nm^2^) shows center-divergent quad-domains with a cross-shaped CDW configuration (Fig. 2a). When an in-plane [$\bar{1}\bar{1}0$]-oriented electric field is applied in the area enclosed by a blue box in Fig. 2a, the pink area of the quad-domain with an in-plane component of [$\bar{1}\bar{1}0$]-oriented polarization grows, but the gray area of the quad-domain with an in-plane component of [$\bar{1}10$]-oriented polarization shrinks. This domain evolution is accompanied by DW bending (Fig. 2b), which drives the local DW to change from a CDW (Fig. 2d) to a neutral DW (Fig. 2e), as demonstrated by the transformation between cross-shaped CDWs and interrupted DWs in the bound charge density map of Fig. 2g and h. The detailed definitions of the CDWs and neutral DWs can be seen in the inserts of Fig. 2d, e, and Supplementary Text. Due to the abrupt change in the bound charge density from a CDW to a neutral DW, as shown in the area enclosed by the blue box in Fig. 2g, h, the conductance is expected to dramatically decrease, which is consistent with the disconnected CDW in Fig. 1e. Intriguingly, if a [$110$]-oriented electric field is applied in the area enclosed by the blue box in Fig. 2b, the disrupted CDW can be reoriented as shown in Fig. 2c, and transforms into a CDW, as demonstrated in Fig. 2f. The recovered high density of the bound charges for the CDW in Fig. 2i explains the reconnected CDWs in Fig. 1f. The corresponding intermediate domain images and the dynamic evolution for the CDWs from Fig. 2a to b, and Fig. 2b to c can also be seen in Supplementary Fig. 12 and Supplementary Movies 1 and 2, respectively. Additionally, we also performed phase-field simulations to investigate the reversible control of one CDW by the alternative [$\bar{1}\bar{1}0$]- and [$110$]-oriented electric field (Fig. S13), which is consistent with the experimental observation in Fig. 1d–f and Fig. S6.

The creation and annihilation of one CDW are related to the variation of the bound charge density during the polarization re-distribution controlled by the external electric field. For the initial state in Fig. 2a, the orientation of the DW marked by the dashed box aligns along $[1\bar{1}0]$-crystalline orientation. In such cases, there are a large number of negative bound charges accumulated at the DW region (marked by ⊖ the symbol in Fig. 2d), which indicates the nano-island with an initial CDW configuration with a high conductance state (Fig. 2g). After the application of [$\bar{1}\bar{1}0$]-oriented electric field, the DW was bent nearly parallel to [$010$]-orientation (Fig. 2b). In this case, the polarization vector rotated continuously across the DW region, resulting in a head-to-tail neutral DW (marked by ⊕ and ⊖ symbols in Fig. 2e), i.e., this segment changes from a CDW to a neutral DW (this segment CDW being annihilated). After applying a reversed electric field ([$110$]-orientation), the DW was bent nearly to [$\bar{1}\bar{1}0$]-orientation (Fig. 2c), where a great number of negative charges (marked by ⊖ symbol in Fig. 2f) accumulated at the DW region once again, resulting in a reversal change of this segment from a neutral DW to a CDW (this segment CDW being re-created, see Fig. 2i).

### Design of NOR and NAND logic gates

**Fig. 3:**
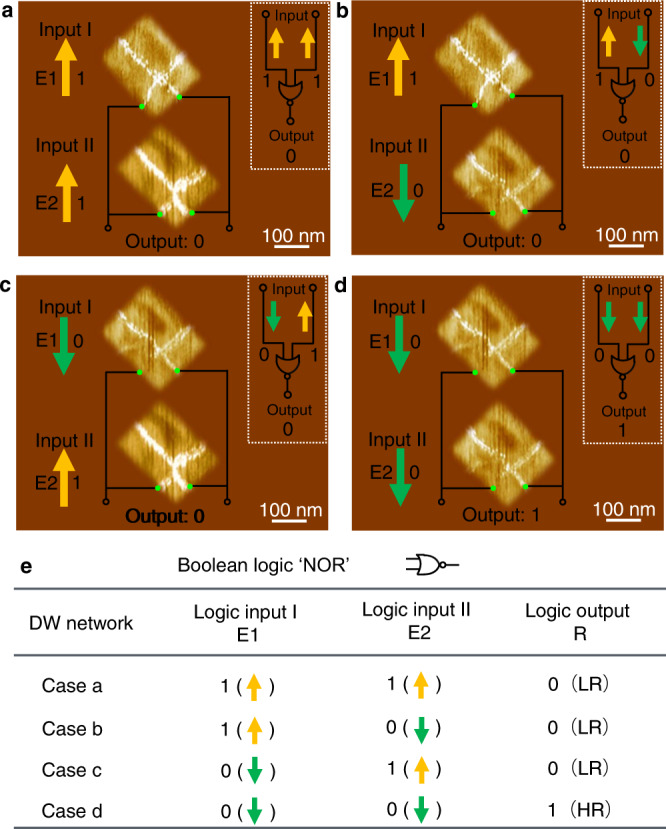
Reconfigurable NOR logic gate. **a**–**d** c-AFM images and corresponding logic circuit diagrams of two parallel-connected nano-islands with the sequence of logic operations for inputs of ‘11’, ‘10’, ‘01’, and ‘00’. **e** Truth table for the NOR logic gate. E1 and E2 are the applied in-plane trailing fields for the two nano-islands.

Based on such robust control characteristics of the cross-shaped CDWs of the off-center vertex domains, more versatile logic gates (e.g., NOR and NAND) can be designed by connecting two rectangular nano-islands in parallel (Fig. 3) or series (Fig. S14). With two nano-islands combined in parallel, as shown in Fig. 3a, the electric fields (E1 and E2) used for control of the CDWs form the two logic inputs, and the conductance state, determined by the parallel resistance of the CDWs confined between the two selected terminals (green nodes), represents the logic output. To illustrate the functionality of the NOR gate, four different logic input configurations of ‘11’, ‘10’, ‘01’, and ‘00’ are illustrated in Fig. 3a–d, where the output conductance state is shown as ‘1’ (high resistance, HR) only when the investigated CDWs of the in-parallel nano-islands are both disconnected; otherwise, it is shown as ‘0’ (low resistance, LR). The relationship between the logic inputs and outputs is summarized in Fig. 3e, which corresponds to the required logic operations for a NOR gate. To further illustrate the working principle of the proposed NOR gate with combined structures, a schematic of the crossbar structure is shown in Fig. S15. The input electric field for the upper panel nano-island is implemented by Word line 1 (WL1) and WL2, and that for the lower panel nano-island is implemented by WL3 and WL4, and the polarity of the electric field is controlled by the switching of the external circuit. The output resistance state is readout by Bit line 1 (BL1) and BL2. With two nano-islands connected in series, a NAND gate is implemented, as shown in Fig. S14. In this case, the output conductance state is shown as ‘0’ (LR) only when the investigated CDWs are both connected; otherwise, it is shown as ‘1’ (HR). Furthermore, the NAND gate can also be implemented when a third nano-island serves as a Bias, as shown in Fig. S16.

### Design of OR, AND, XOR, and XNOR logic gates

**Fig. 4:**
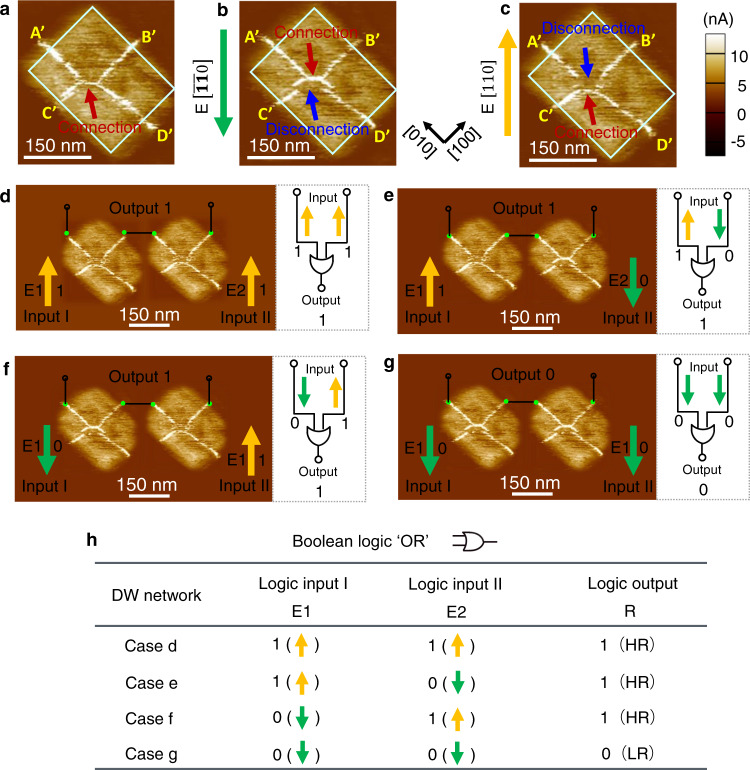
Reconfigurable OR logic gate. **a**–**c** CDW networks at the initial state and controlled by an in-plane trailing field in a BiFeO_3_ nano-island with an AR of 1.25. The connection/disconnection of ${A}^{{\prime} }$/${B}^{{\prime} }$ node and disconnection/connection of ${C}^{{\prime} }$/${D}^{{\prime} }$ node is implemented concurrently in one nano-island by applying an in-plane field along [$\bar{1}\bar{1}0$] (**b**) or [$110$] direction (**c**). **d**–**g** c-AFM images and corresponding logic circuit diagrams of two series-connected nano-islands with the sequence of logic operations for inputs of ‘11’, ‘10’, ‘01’, and ‘00’. **h** Truth table for OR logic gate respectively. E1 and E2 are the applied  in-plane trailing fields for the two nano-islands. R represents the resistance of the DW between the output electrodes.

According to the ‘NOT’ logic gate illustrated in Fig. 1d–f, it is challenging to implement ‘1’ and ‘0’ logic outputs when the logic inputs are ‘11’ and ‘00’, respectively. While these logic operations are the essential ingredients for other basic logic gates, such as ‘OR’, ‘AND’, ‘XOR’, and ‘XNOR’. Thus, more versatile CDWs are needed to be explored, where the connection of one CDW and disconnection of another CDW are required concurrently when a unidirectional trailing field along [$\bar{1}\bar{1}0$] or [$110$]-orientation is applied on one nano-island. Accordingly, we explore the modulation of CDWs in a nano-island with the size of 260 × 208 nm^2^ and AR of 1.25) as shown in Fig. 4a–c, where the initial four quad-domains show different symmetry compared with that of the above discussed four quad-domains. As can be seen in Fig. 4a, the c-AFM image shows ~20 nA conductance at the CDW locations when the polarizations are upward and center-divergent. The modulated CDWs by in-plane trailing field are shown in Fig. 4b, c. When the in-plane trailing field is along [$\bar{1}\bar{1}0$] direction, we observe that two-quarters of the DWs at the lower/upper panel are disconnected/connected (Fig. 4b), while what happens for the two-quarters of the DWs at the upper/lower panel when the in-plane trailing field is along [$110$] direction (Fig. 4c). Thus, the high and low conductance states can be simultaneously implemented between ${A}^{{\prime} }$/${B}^{{\prime} }$ node and ${C}^{{\prime} }$/${D}^{{\prime} }$ node of the CDW network in Fig. 4b, as well as ${C}^{{\prime} }$/${D}^{{\prime} }$ node and ${A}^{{\prime} }$/${B}^{{\prime} }$ node in Fig. 4c, respectively. Taking advantage of such CDWs controlled by in-plane downward and upward trailing fields in Fig. 4b, c, we design ‘OR’ and ‘AND’ logic gates by series-connected and parallel-connected two nano-islands with changeable DW networks as discussed above respectively, as shown in Fig. 4d–h and Fig. S17. In addition, other logic gates such as ‘XOR’ and ‘XNOR’ are also designed as shown in Figs. S18 and S19, respectively.

### Digital signal readout and transmission by ferroelectric DW logic circuits

**Fig. 5:**
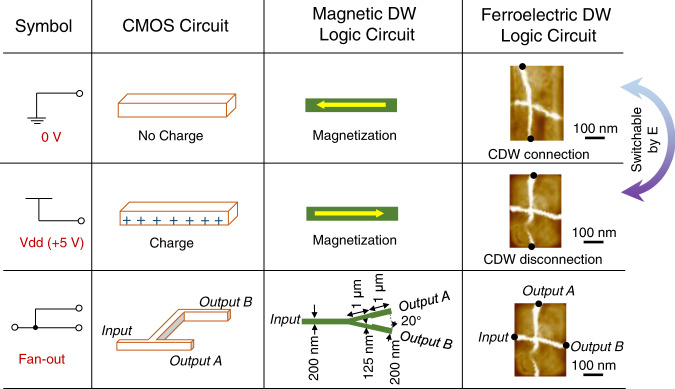
Electric field control of ferroelectric CDW logic circuits. Schematic illustration of the ferroelectric DW logic circuits for the readout signal of low and high voltages and the fan-out function. The bidirectional arrow indicates that the readout signals of low and high voltages can be transformed into each other by electric-field-induced connection and disconnection of a CDW. The schematics for the CMOS circuit and magnetic DW logic circuit, redrawn from ref. ^8^ are also illustrated for comparison. E represents the applied in-plane electric field.

The demonstrations of the above logic gates make the concept of ferroelectric DW logic viable since any Boolean function can be implemented by combining these basic gates. Furthermore, signal readout and transmission, as schematically shown in the first column of Fig. 5, are also proposed based on an electric-field-controllable ferroelectric CDW network. For comparison, complementary metal-oxide semiconductor (CMOS) and ferromagnetic DW logic circuits^8^ are also highlighted, as shown in the second and third columns of Fig. 5, respectively. For the signal readout, a low voltage and a high voltage can be read out directly at two ends (marked by black points) of a connected (the first panel of the fourth column in Fig. 5) and disconnected (the second panel of the fourth column in Fig. 5) CDW, and the readout signals can be easily switched between each other by an electric field (indicated by the bidirectional arrow in Fig. 5). The third panel of the fourth column of Fig. 5 shows a fan-out circuit, where an input signal can be separated into two output signals of A and B. In this way, one might be able to integrate all-electric logic gates and circuits into the nano-island array and regulate the function of each specific logic unit as needed.

## Discussion

As discussed at the beginning, in comparison with emerging designs of racetrack memory and DW logic based on moving magnetic DW^6–9^, the ferroelectric DW logic units exhibit higher integration density (Fig. S20) due to much smaller DW width and lower energy consumption due to electric-field-driven features. More comparison in the Supplementary Table 1 demonstrates some benefits of the ferroelectric DW logic circuit for device applications, such as low energy consumption, fast operation speed, and high integration density. For example, the operating energy for one bit estimated for the ferroelectric DW logic units is in 10 aJ order of magnitude, which is comparable to the emerging energy-efficient magnetoelectric spin-orbit logic but much lower than other emerging magnetic and existing CMOS technologies^36^. Finally, it should be noted that the proposed ferroelectric DW logic units are preliminary designs and there are several challenges. For example, the endurance of the CDW network under electric field-driven repeated on-and-off switching (Figs. 1d–f) and operation at higher temperatures (Fig. S9) remain to be improved. One possible way to address this issue in the future is to design the geometry (size and AR) of the nano-islands^30,37^, such that the shape and thickness of the CDW might be accurately confined by the symmetry of the nano-islands. Another big challenge is the logic cascading of the single logic gates, which may be implemented by appropriate circuit design and connection, e.g., the voltage corresponding to the resistance state of the DW can be set as output, and then the output voltage can be used as the input of the next logic gate, to achieve cascade.

In summary, we demonstrate electric-field controlled stable and repeatable on-and-off switching of conductive domain walls within topologically confined vertex domains naturally formed in self-assembled ferroelectric nano-islands, which is accompanied by the transformations between charged and neutral domain walls via electric-field controlled domain-wall reconfiguration. By analogous to logic processing, we also propose programmable logic gates and circuits based on reconfigurable conductive domain walls. This ferroelectric memory-in-logic architecture proposed based on the flexible control of conducting domain-wall network might allow for designing programmable all-electric logic with low energy consumption.

## Methods

### Preparation of ferroelectric nano-island array with CDWs

BiFeO_3_ thin films were prepared by pulsed laser deposition method on LaAlO_3_ (001) single-crystal substrates, where a very thin (2 nm) (La,Sr)MnO_3_(LSMO) buffer layer was pre-grown to construct the required electrostatic and elastic boundary conditions for the nucleation of self-assembled BiFeO_3_ nano-islands. During the thin-film preparation, the growth temperature was maintained in the range of 600–750 °C under the oxygen pressure of 0.2 mbar. The growth details for BiFeO_3_ nano-island array are similar to our previous work^27^.

### PFM measurements for ferroelectric-domain configurations

The polar vectors of the three-dimensional BiFeO_3_ nano-islands were investigated by the combination of vertical and lateral PFM phase images using an Infinity Asylum Research AFM and a Bruker AFM. During the measurement, a commercial Pt/Ir-coated tip with a tip radius of 20 nm, a force constant of 2.8 N/m, and a resonant frequency of 75 kHz was used as a movable top electrode, where the bottom electrode (LSMO layer) was ground. The PFM signal was collected at the contact resonance frequency with an a.c. tip bias of 1 V_pp_, and the scanning velocity of the cantilever is 0.5 μm/s.

### c-AFM measurements for conducting DW network

The conductance of the BiFeO_3_ nano-islands was characterized by c-AFM, which is also based on an Infinity Asylum Research AFM and a Bruker AFM. In the c-AFM model, a commercial Pt/Ir-coated tip was used. During the conductance measurement, the tip was ground and the voltage bias was applied to the LSMO bottom electrode. The bias voltage applied to the LSMO bottom electrode is ~1.5 V.

### Phase-field simulations

Phase-field simulations were carried out using the time-dependent Ginzburg-Landau model for the investigation of domain evolution and DW network in BiFeO_3_ nano-islands. Ferroelectric polarization evolution is described by the polarization vector ${{{{{{\bf{P}}}}}}}_{i}$(${P}_{x}$, ${P}_{y}$, ${P}_{z}$), which is the order parameter to describe the domain structure. The model describes the temporal evolution of domain structure, which can be numerically solved by the time-dependent Landau-Ginzburg (TDGL) equation,

1 $$\frac{\partial {{{{{{\bf{P}}}}}}}_{i}({{{{{\bf{r}}}}}},t)}{\partial t}=-L\frac{\delta {F}_{p}}{\delta {{{{{{\bf{P}}}}}}}_{i}\left({{{{{\bf{r}}}}}},t\right)},\left(i=x,y,z\right)$$

where *t*, *L*, and *F*_*p*_ are simulation time, kinetic coefficient (related to DW mobility), and total free energy respectively, whereas:

2 $${F}_{P}=\mathop{\iiint}\limits_{V}\big({f}_{{{\mbox{bulk}}}}\left({{{{{{\bf{P}}}}}}}_{i}\right)+{f}_{{{\mbox{grad}}}}\left({{{{{{\bf{P}}}}}}}_{i,j}\right)+{f}_{{{\mbox{elas}}}}\left({{{{{{\bf{P}}}}}}}_{i,}{\epsilon }_{{ij}}\right)+{f}_{{{\mbox{elec}}}}({{{{{{\bf{P}}}}}}}_{i,}{{{{{{\mathbf{E}}}}}}}_{i}){dV}$$

where *f*_bulk_, *f*_grad_, *f*_elas_, and *f*_elec_ are bulk free energy density^38^, gradient energy density, elastic energy density, and electric energy density^39^, respectively. The bulk free energy density *f*_bulk_ is a sixth-order polynomial,

3 $${f}_{{{\mbox{bulk}}}} =	 \,{a}_{1}\left({P}_{x}^{2}+{P}_{y}^{2}+{P}_{z}^{2}\right)+{a}_{11}\left({P}_{x}^{4}+{P}_{y}^{4}+{P}_{z}^{4}\right)+{a}_{12}\left({P}_{x}^{2}{P}_{y}^{2}+{P}_{y}^{2}{P}_{z}^{2}+{P}_{z}^{2}{P}_{x}^{2}\right)\\ 	+{a}_{111}\left({P}_{x}^{6}+{P}_{y}^{6}+{P}_{z}^{6}\right)+{a}_{112}({P}_{x}^{4}({P}_{y}^{2}+{P}_{z}^{2})+{P}_{y}^{4}({P}_{z}^{2}+{P}_{x}^{2})+{P}_{z}^{4}({P}_{x}^{2}+{P}_{y}^{2}))+{a}_{123}{P}_{x}^{2}{P}_{y}^{2}{P}_{z}^{2}$$

where ${\alpha }_{1},{\alpha }_{11},{\alpha }_{12},{\alpha }_{111},{\alpha }_{112}$ and ${\alpha }_{123}$ are dielectric stiffness and high order stiffness. Among them, only ${\alpha }_{1}$ depends on the temperature, such that ${\alpha }_{1}$ = (*T*−*T*_0_)/(2*ε*_0_C_0_), *T* is temperature, *T*_0_ is Curie temperature, *ε*_0_ = 8.85 × 10^−12 ^F/m is dielectric permittivity of vacuum, *C*_0_ is the Curie constant.

The gradient energy density is described in terms of polarization gradients. For simplicity, the gradient energy is taken to be isotropic, given as:

4 $${f}_{{{\mbox{grad}}}}=\frac{1}{2}{g}_{{ijkl}}{{{{{{\bf{P}}}}}}}_{i,j}$$

where the ${g}_{{ijkl}}$ is gradient energy coefficient and ${{{{{{\bf{P}}}}}}}_{i.j}=\partial {P}_{i}/\partial {x}_{j}$^40^$.$ The elastic energy can be written as,

5 $${f}_{{{\mbox{elas}}}}=\frac{1}{2}{c}_{{ijkl}}{e}_{{ij}}{e}_{{kl}}=\frac{1}{2}{c}_{{ijkl}}\left({\varepsilon }_{{ij}}-{\varepsilon }_{{ij}}^{0}\right)\left({\varepsilon }_{{kl}}-{\varepsilon }_{{kl}}^{0}\right)$$

where the ${c}_{{ijkl}}$ is elastic stiffness tensor, ${e}_{{ij}}$ is elastic strain, ${\varepsilon }_{{ij}}$ is total elastic strain, ${\varepsilon }_{{ij}}^{0}$ is the stress-free strain given as ${\varepsilon }_{{ij}}^{0}={Q}_{{ijkl}}{P}_{k}{P}_{l}$, where ${Q}_{{ijkl}}$ represents the electrostrictive coefficient^41^. The electrostatic energy, ${f}_{{{\mbox{elec}}}}$, is given by,

6 $${f}_{{{\mbox{elec}}}}={-{{{{{\bf{P}}}}}}}_{i}{{{{{{\bf{E}}}}}}}_{i}-\frac{{\varepsilon }_{0}{\varepsilon }_{r}}{2}{{{{{{\bf{E}}}}}}}_{i}{{{{{{\bf{E}}}}}}}_{i}$$

In the present simulations, an open circuit boundary condition with fully compensated edge charges (156 μC/cm^2^) on edge tilts and charge-free on the substrate and top surface was used as an electrostatic boundary condition, and a strain-free mechanical boundary condition was used at the working temperature of 300 K. The nano-island was constructed with a size of 178 nm × 138 nm at the base and 7 nm at the island height, and the edge tilt was taken as 45° for the simulations. The value of coefficients used for the current work is listed in Supplementary Table 2^42^. The parameters of permittivity and Curie constant (*C*_0_) used in the phase-field simulations are 50 and 1.2166 × 10^5 ^K, respectively.

### Reporting summary

Further information on research design is available in the Nature Research Reporting Summary linked to this article.

## Supplementary information

Supplementary Information

Description of Additional Supplementary Files

Supplementary Movie 1

Supplementary Movie 2

Reporting Summary

Lasing Reporting Summary

## Data Availability

The authors declare that the data supporting the findings of this study are available within the paper and its Supplementary Information Files.
