# Supplementary material for: Ferroelectric domain-wall logic units
Source: Nat Commun. 2022 Jun 6;13:3255. doi: 10.1038/s41467-022-30983-4 (PMC9170692; doi:10.1038/s41467-022-30983-4)
Supplement: Supplementary file 1 — Supplementary Information [file 41467_2022_30983_MOESM1_ESM.doc]

Supplementary Materials for

**Ferroelectric domain-wall logic units**

Jing Wang^1,2#^, Jing Ma^2#^, Houbing Huang^1*^, Ji Ma^2,3^, Hasnain Mehdi Jafri^1^, Yuanyuan Fan^1^, Huayu Yang^1^, Yue Wang^2^, Mingfeng Chen^2^, Di Liu^1^, Jinxing Zhang^4^, Yuan-Hua Lin^2^, Long-Qing Chen^5^, Di Yi^2^, Ce-Wen Nan^2*^

^1^Advanced Research Institute of Multidisciplinary Science, and School of Materials Science and Engineering, Beijing Institute of Technology; Beijing 100081, China

^2^State Key Laboratory of New Ceramics and Fine Processing, School of Materials Science and Engineering, Tsinghua University; Beijing 100084, China

^3^School of Material Science and Engineering, Kunming University of Science and Technology; Kunming, Yunnan 650093, China

^4^Department of Physics, Beijing Normal University; Beijing 100875, China

^5^Department of Materials Science and Engineering, Pennsylvania State University; University Park, PA 16802, USA

#These authors contributed equally to this work.

Correspondence to: [hbhuang@bit.edu.cn](mailto:hbhuang@bit.edu.cn); [cwnan@mail.tsinghua.edu.cn](mailto:cwnan@mail.tsinghua.edu.cn)

This PDF file includes:

Supplementary Text

Supplementary Figures 1 to 20

Supplementary Tables 1, 2

Other Supplementary Materials for this manuscript include the following:

Supplementary Movies 1, 2

Supplementary Text

**Creation of an in-plane trailing field by the biased scanning probe.**

As shown in Supplementary Figure 5, the spatial distribution of the static electric field from the nanoscale probe is isotropic and spherical. As shown in the schematic of Supplementary Figure 5a, the fast-scanning direction of the nanoscale probe (negative biased) is alternative from left to right and from right to left, while the slow-scanning direction is either in-plane downward or upward. When the slow-scanning direction for the negative-biased probe is from up to down, the resulting in-plane electric field, namely the trailing field, is in the direction from up to down (Supplementary Figure 5b). While, when the slow-scanning direction is from down to up, the induced in-plane trailing field is in the direction from down to up (Supplementary Figure 5c). This bi-polar trailing field created by the biased scanning probe is consistent with the results reported in the previous literature [1-3]. It is notable that during the CDW network evolution, the in-plane trailing field from the biased probe makes the major contribution, but the vertical component of the electric field does not, because both the out-of-plane polarization projection of the quad-domain and the vertical field are upward.

**The definition of CDWs and neutral DWs.**

To illustrate the microscopic mechanism of the various conductance of DWs with distinct DW morphology, we analyzed the distribution of the polarization vectors and the bound charge density around the local DWs. As shown in Fig. 2d and 2f, when the DW is oriented along $[1\bar{1}0]$ or $[\bar{1}\bar{1}0]$ direction respectively, there are a large number of bound charges accumulated on the DW, which is defined as CDW. While in Fig.2e, the DW is oriented almost along [010] direction, which results in a continuous transition of the polarization vectors and dramatically reduction of the bound charge density at such a DW. Hence, such kind of DW is defined as neutral DWs. And the above discussions are also well consistent with the high- and low-conductance of the respective DWs, as indicated by the c-AFM snapshots in Fig. 1. Therefore, the above results show clearly that the conducting DW network can be well determined by the DW morphology.

**Comparison of ferroelectric DW logic units with emerging magnetic and the existing CMOS techniques.**

(1) *Low energy consumption.* Both the writing and readout of the signal are through electric voltage, which is superior to the present magnetic domain-wall racetrack memory [4] or logic [5] due to their current writing/readout. The current density required to move magnetic DW and reversal DW chirality is ~10^11^-10^12^ A/m^2^, which indicates high energy consumption. To make a quantitative comparison, we estimate the energy consumption for one-bit operation in the proposed ferroelectric CDW logic. Considering the operating voltage U=3 V, the resistance of DW R~1.6 GΩ, and the operating time of t < 1ns [6, 7], the operating energy for one bit is$\frac{U^{2}}{R}t$~3.9 aJ, which is similar to the proposed energy-efficient magnetoelectric spin-orbit logic and about 10-30 times below that of the advanced CMOS technique [8]*.* The comparison of the energy consumption for one-bit operation is summarized in Supplementary Table 1.

Supplementary Table 1. Comparison of the energy consumption for multiple devices

| Devices | Write energy (per bit) |
| --- | --- |
| Flash-NAND | 1 nJ [9] |
| FeRAM | 50 fJ [9] |
| MRAM | 70 pJ [10] |
| STT-RAM | 0.1 pJ [10] |
| SME-RAM | 1.6×10^-4^ pJ [10] |
| MESO-logic | 1-10 aJ [8] |
| Magnetic DW-logic | ~ 20 pJ [5] |
| Ferroelectric DW-logic | ~3.9 aJ |

(2) *High integration density.* Considering the reconfigurable DW configuration in such a small nano-island, we can achieve a NOT gate in 0.3 × 0.2 μm^2^, which is 13 factors lower than the 0.8 × 1 µm^2^ of NOT gate in magnetic DW logic [5]. This critical size can be further decreased to ~ 0.13 × 0.10 μm^2^ according to the simulation results in Supplementary Figure 20. More importantly, considering the CDW width of 3-5 nm, we can achieve a contact of 10 nm, which is similar to that of the new proposed magnetoelectric spin-orbit logic [8].

(3) *Fast operation speed*. Considering the ultrafast switching ferroelectric polarization (sub-ns) [6, 7], this ferroelectric DW logic can be ultrafast devices, much faster than 10-20 ns for the present MRAM, STT-RAM, etc. [10].

Supplementary Figures:


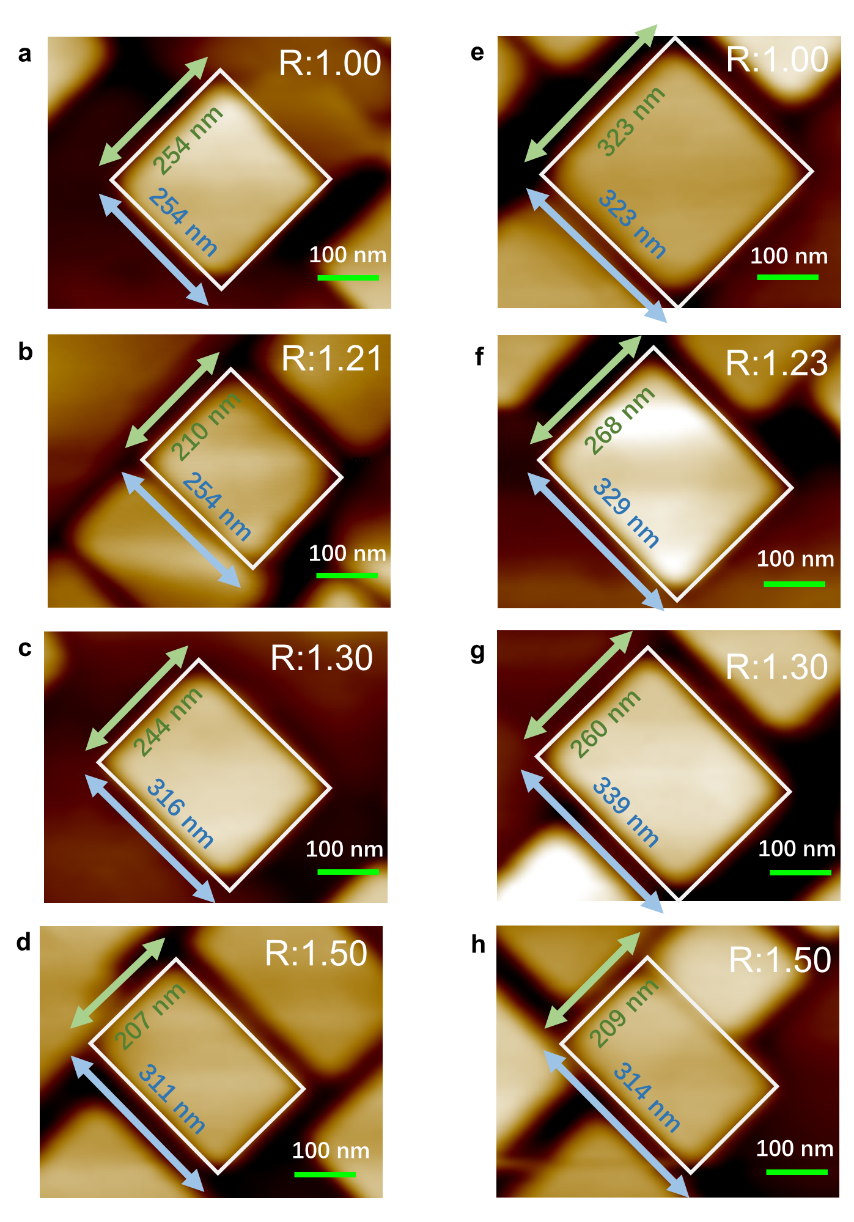


Supplementary Figure 1. Self-assembled BiFeO_3_ nano-islands with various sizes and ARs.


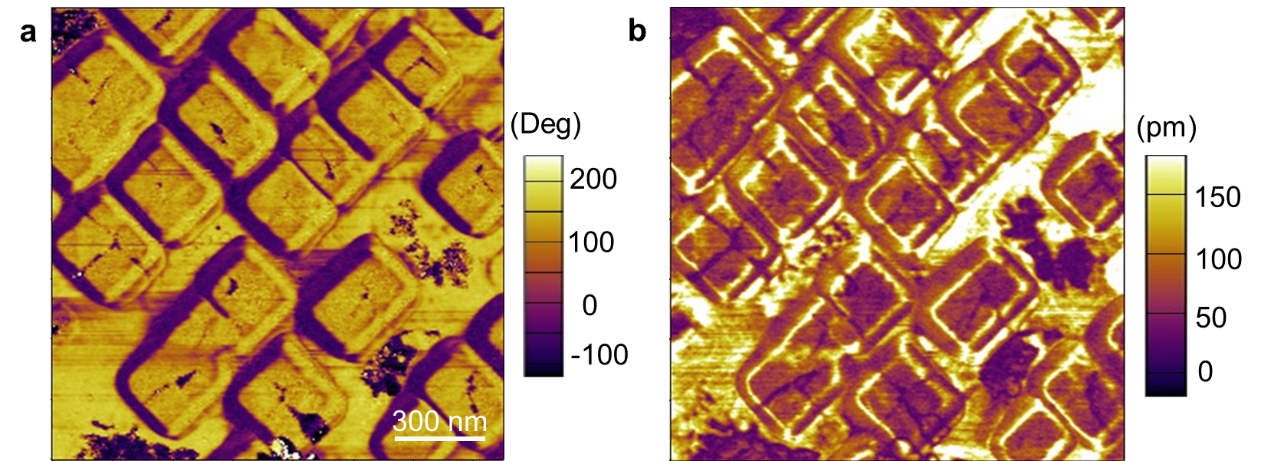


Supplementary Figure 2. Cross-shaped CDW network confined in BiFeO_3_ nano-islands with different sizes and ARs. **a**, Out-of-plane PFM phase image. **b**, Out-of-plane PFM amplitude image.


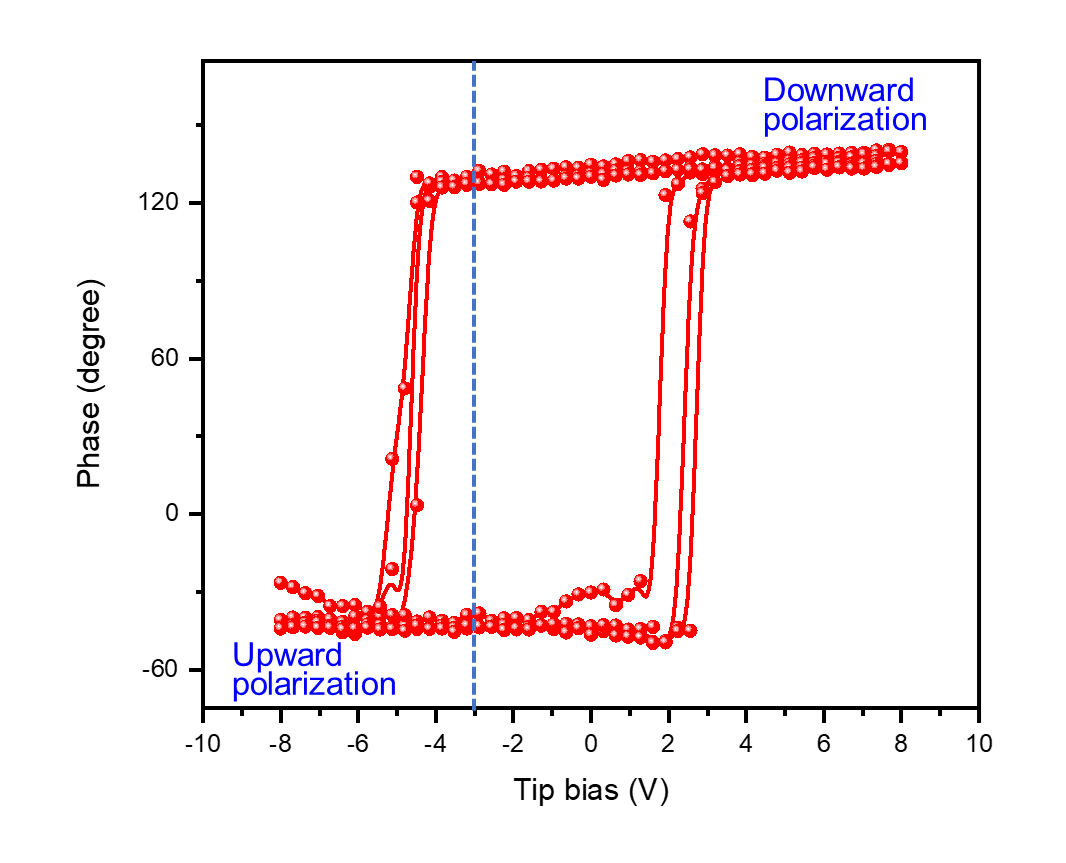


Supplementary Figure 3. Ferroelectric hysteresis loops obtained from the quad-domain region in BiFeO_3_ nano-island.


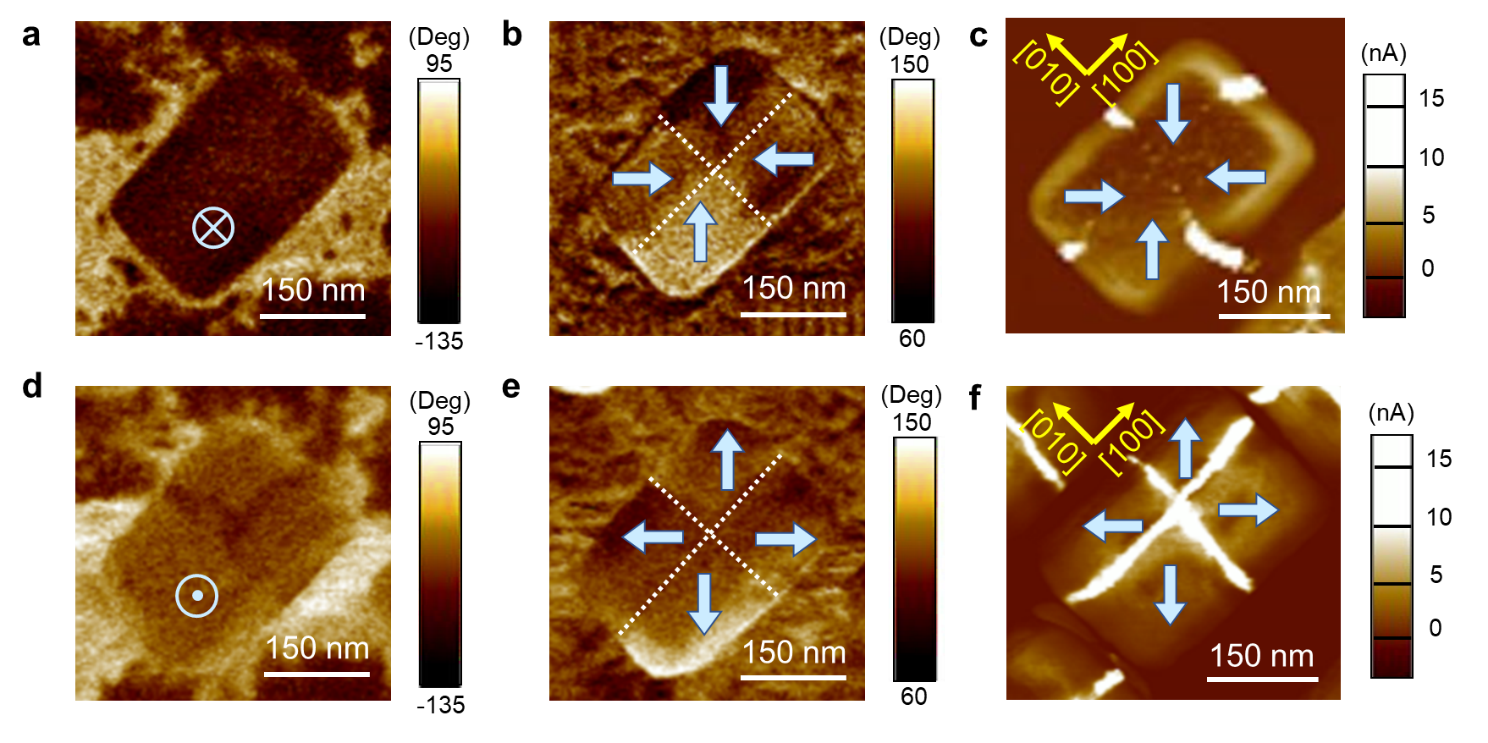


Supplementary Figure 4. Polarization-dependent CDW conduction in a BiFeO_3_ nano-island. **a**-**c**, Out-of-plane (**a**), in-plane (**b**) PFM phase image, and c-AFM image (**c**) for a BiFeO_3_ nano-island with downward and center-convergent domain pattern. **d**-**f** Out-of-plane (**d**), in-plane (**e**) PFM phase image, and c-AFM image (**f**) for the BiFeO_3_ nano-island with upward and center-divergent domain pattern.


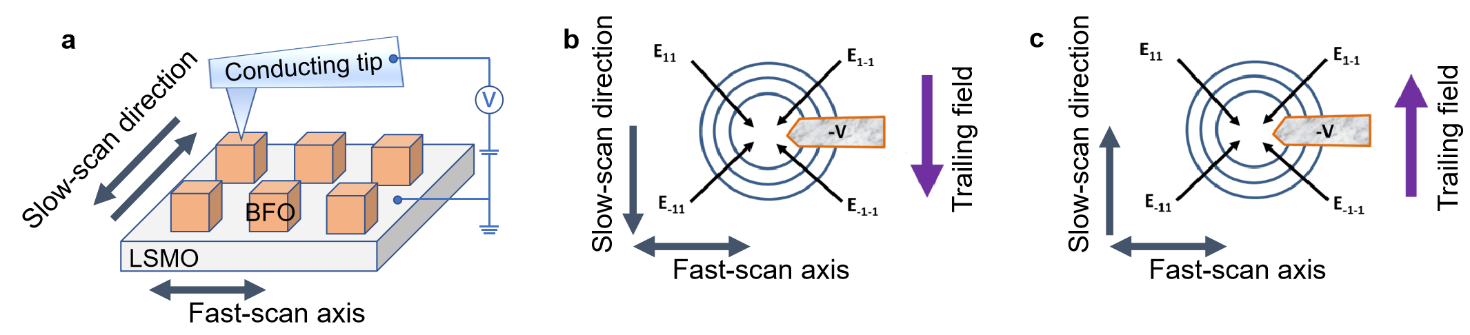


Supplementary Figure 5. Schematic illustration for a trailing field from the negatively biased scanning probe. **a** Schematic diagram of the experimental setup for electric-field control of DW networks. **b**, **c** In-plane electric-field distribution for the moving probe from top to down and down to the top, respectively. The black center-convergent arrows indicate an in-plane radial electric field distribution for the negatively biased probe. The blue contour hints at the equipotential lines. The purple arrows indicate the direction of the trailing field.


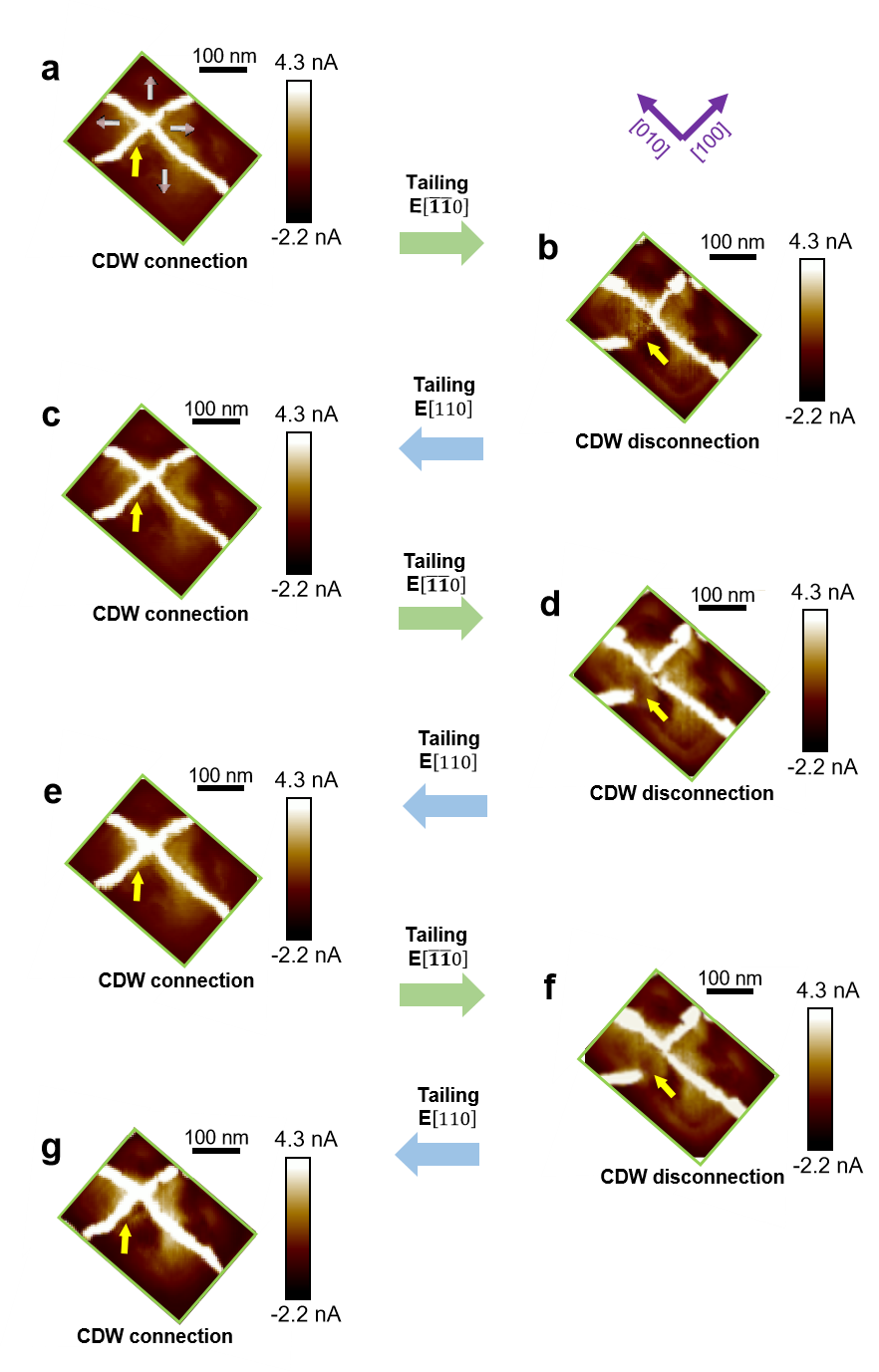


Supplementary Figure 6. Switching endurance for the CDW network by the alternative [$110$]- (marked by the blue arrow) and [$\bar{1}\bar{1}0$]-oriented (marked by green arrow) trailing field.


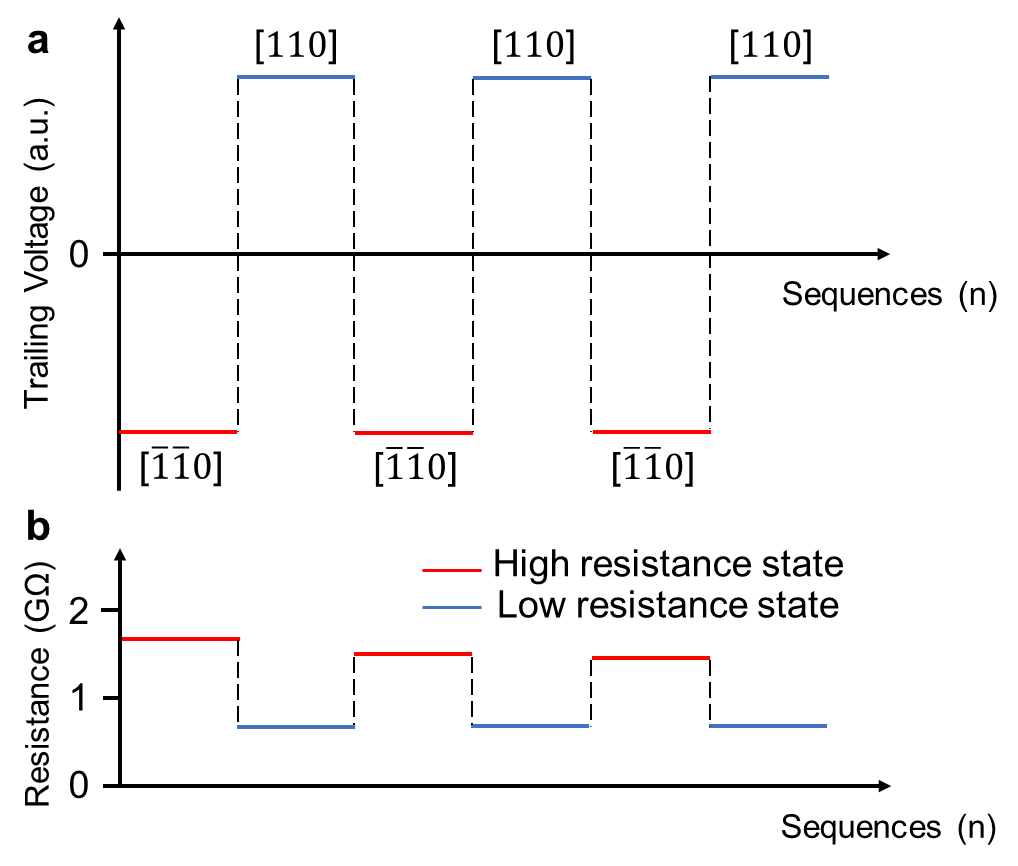


Supplementary Figure 7. The resistance state of CDW switched by alternative [$\bar{1}\bar{1}0$]- and [$110$]-oriented electric field.


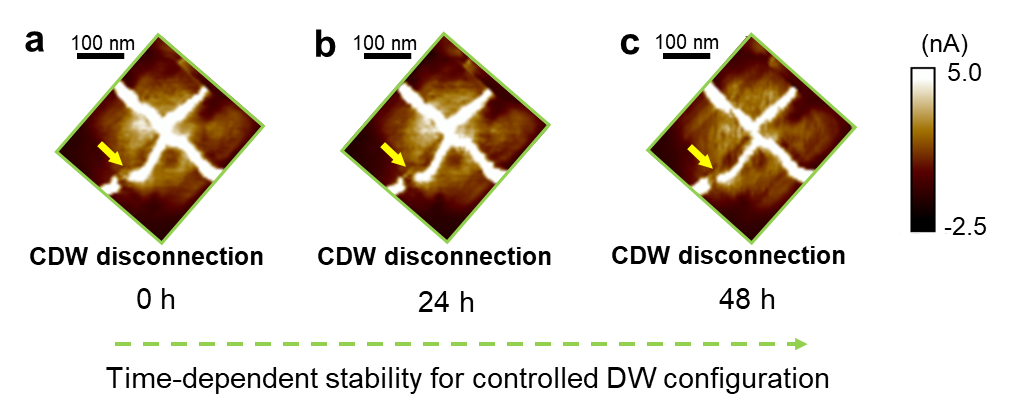


Supplementary Figure 8. Time-dependent retention effect for the controlled CDW network.


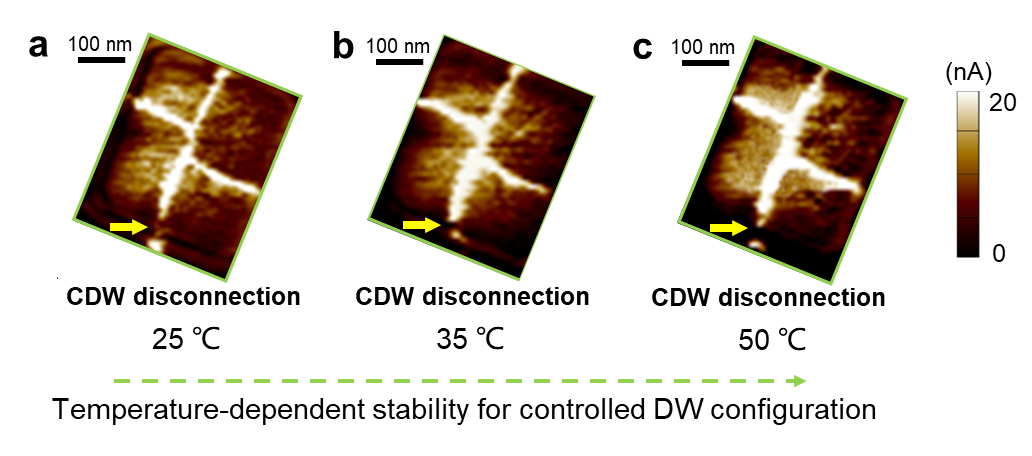


Supplementary Figure 9. Temperature-dependent retention effect for the controlled CDW network.


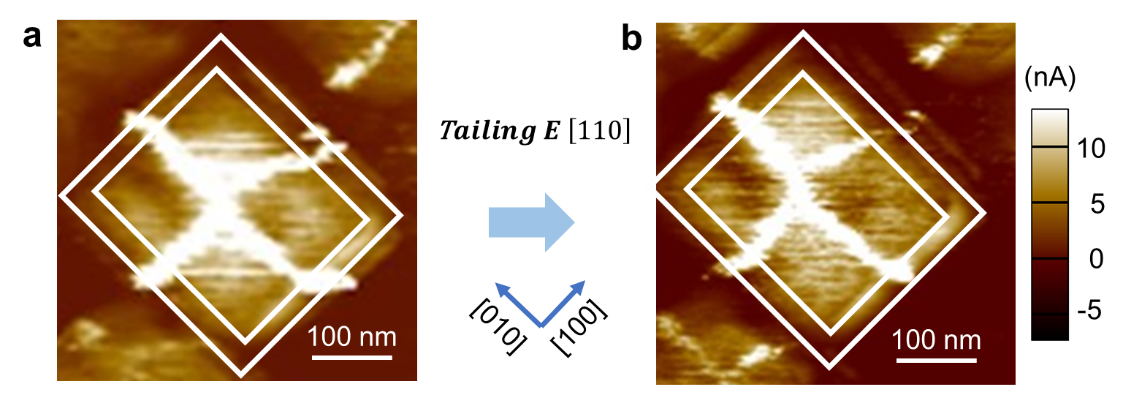


Supplementary Figure 10. Virtually unchanged cross-shaped CDW network under a trailing field in a nano-island with the AR around 1.0.


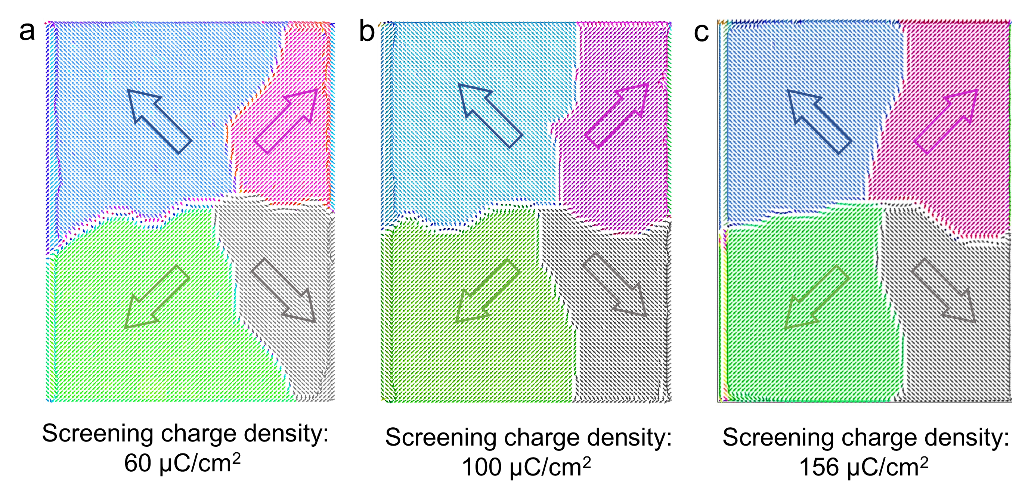


Supplementary Figure 11. Phase-field simulation of quad-domain structure with different screening charge density.

Supplementary Figure 12. The DW evolution during the disconnection (a-d) and reconnection (e-h) process driven by [$110$]- and [$\bar{1}\bar{1}0$]-oriented electric field, respectively.


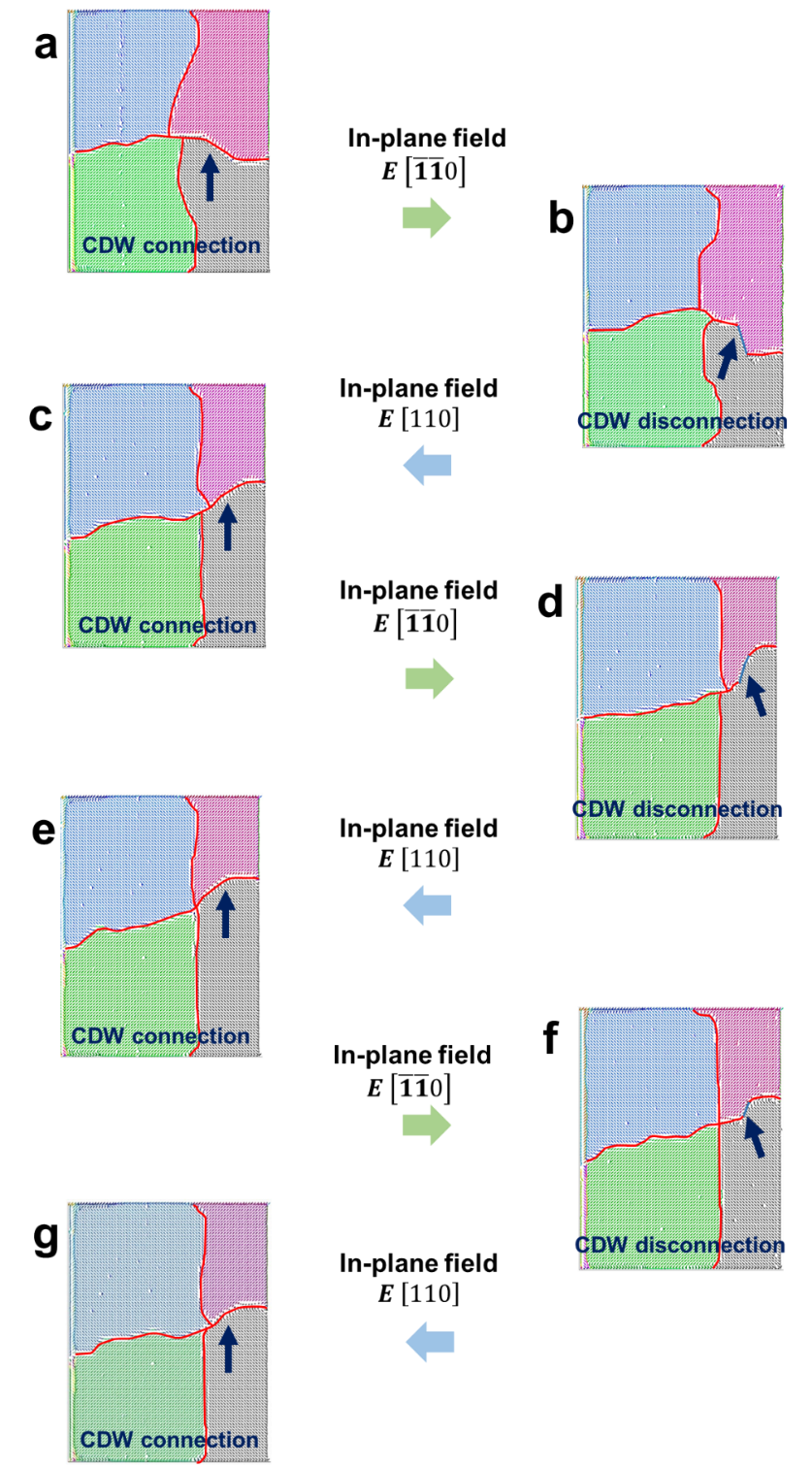


Supplementary Figure 13. Reversible connection and disconnection of one branch of CDW performed by phase-field simulations.


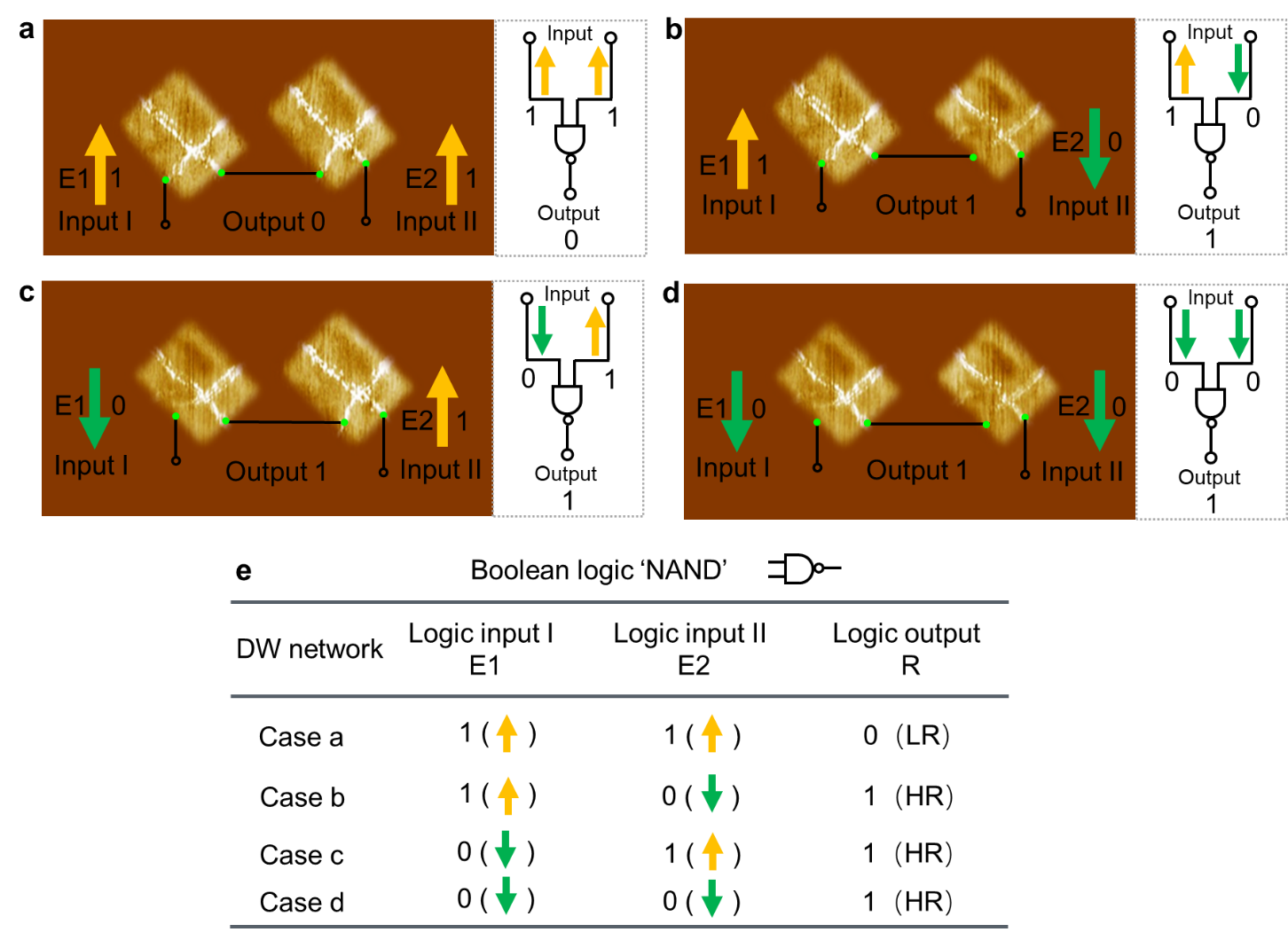


Supplementary Figure 14. Reconfigurable NAND logic gate. **a**-**d**, c-AFM images and corresponding logic circuit diagrams of two series-connected nano-islands with the sequence of logic operations for inputs of ‘11’, ‘10’, ‘01’, and ‘00’. **e**, Truth table for the NAND logic gate.


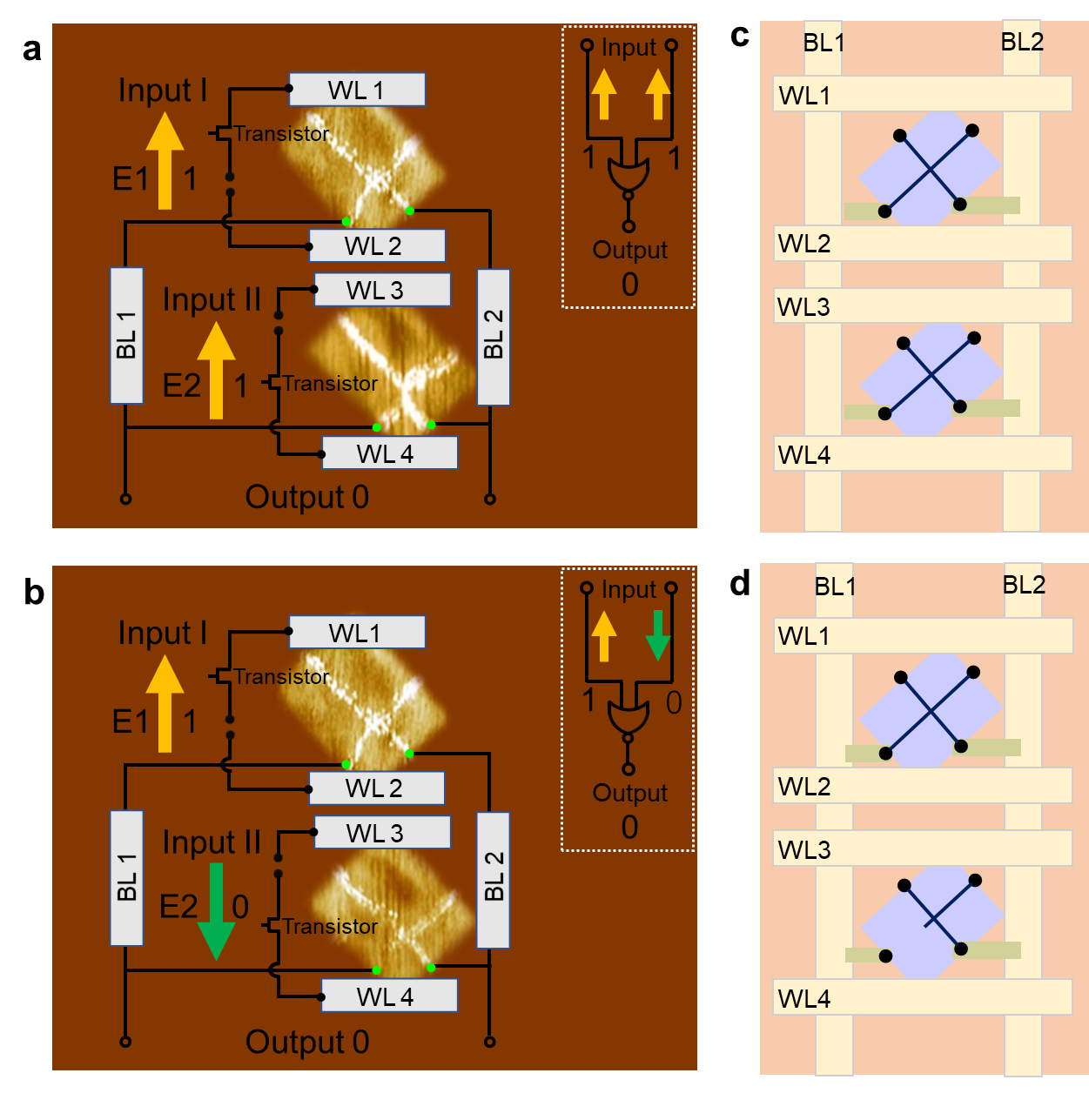


Supplementary Figure 15. Schematic diagram for implementing the ‘NOR’ gate.


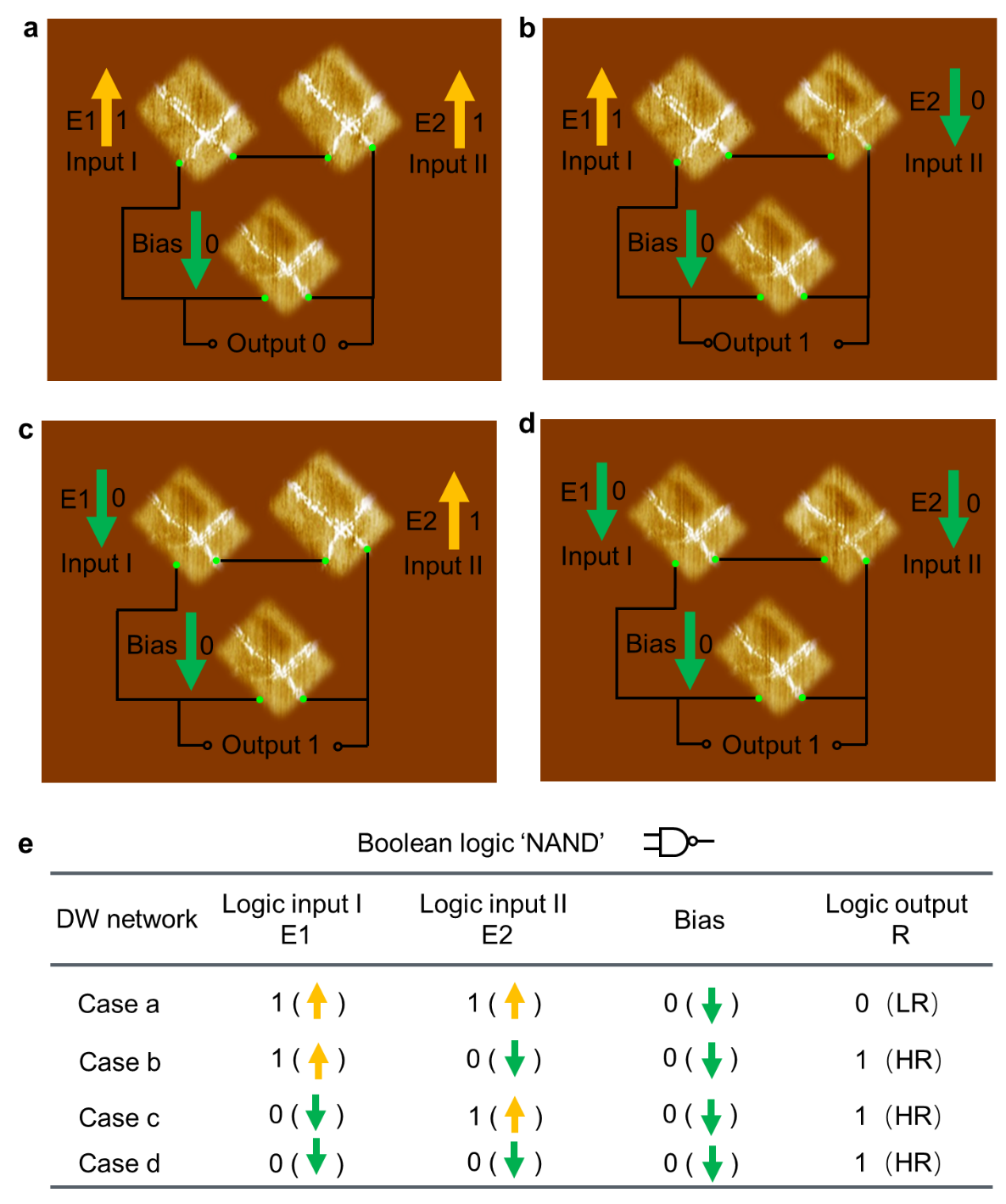


Supplementary Figure 16. Reconfigurable NAND logic gate combined with a Bias.


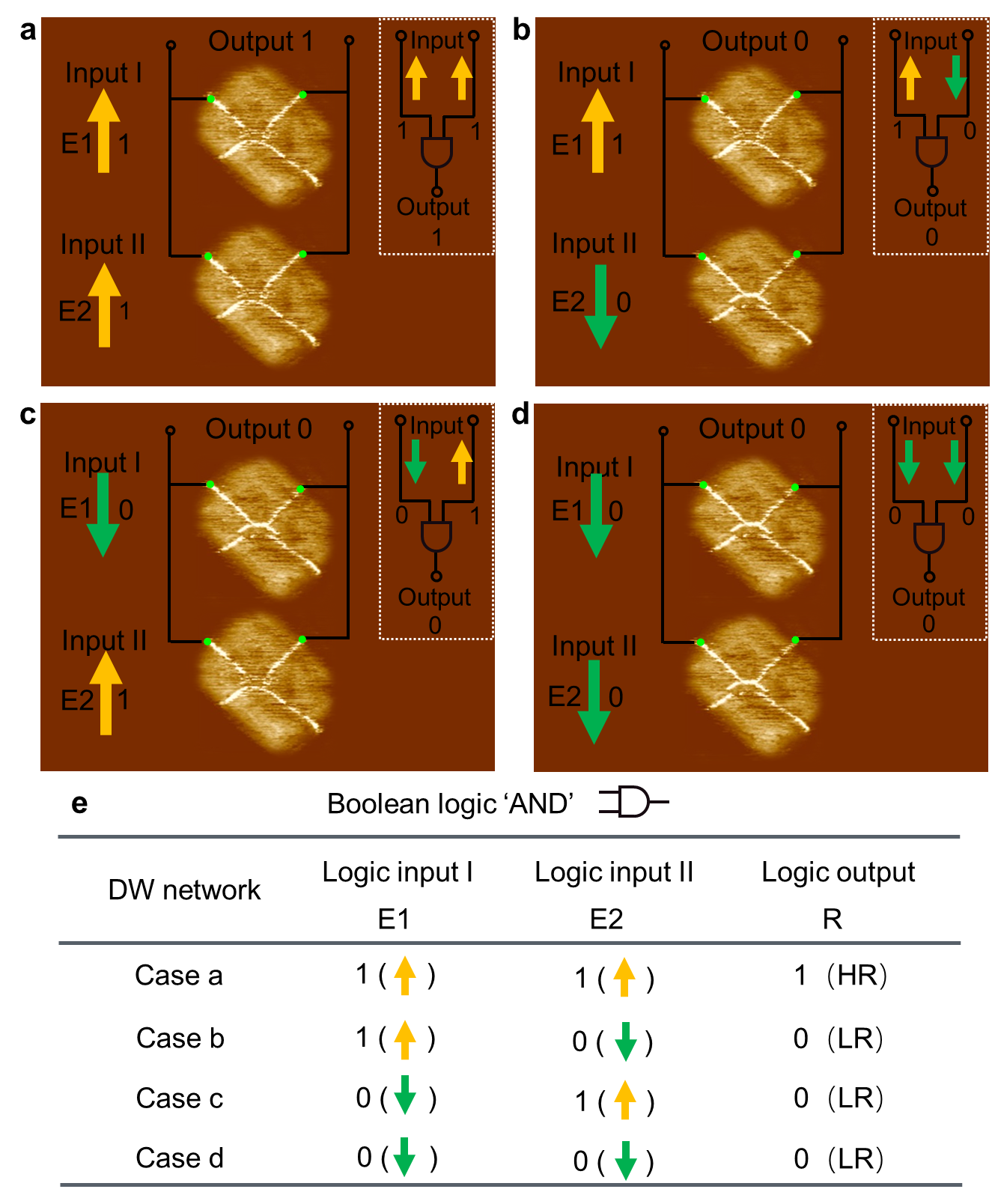


Supplementary Figure 17. Reconfigurable AND logic gate. **a**-**d**, Schematics of different logic operations for inputs of ‘11’, ‘10’, ‘01’, and ‘00’ when two nano-islands are joined in parallel, respectively. **e**, Truth table for AND logic gate.


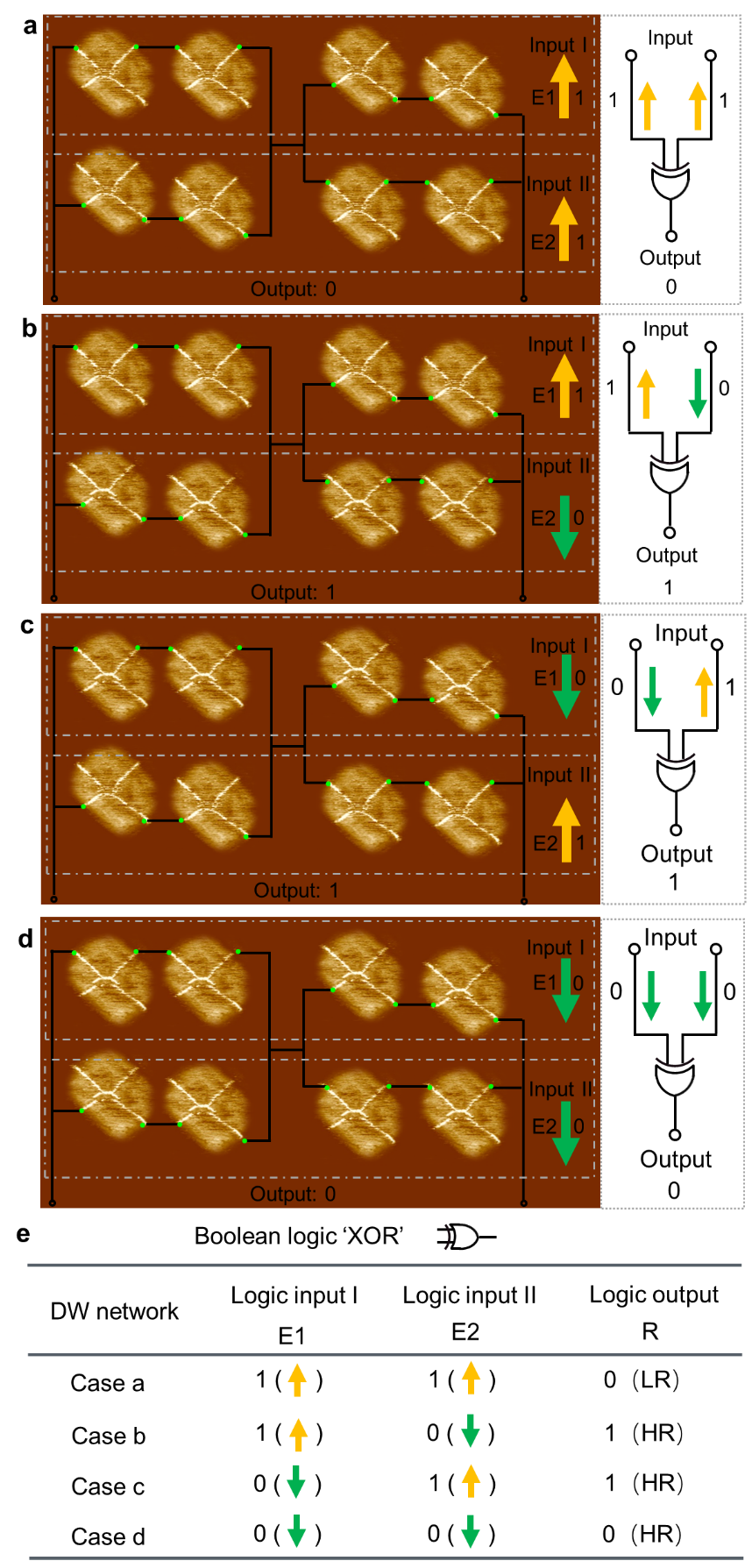


Supplementary Figure 18. Reconfigurable XOR logic gate. **a**-**d**, Schematics of different logic operations for inputs of ‘11’, ‘10’, ‘01’, and ‘00’ when multiple nano-islands are joined in series and parallel. **e**, Truth table for XOR logic gate.


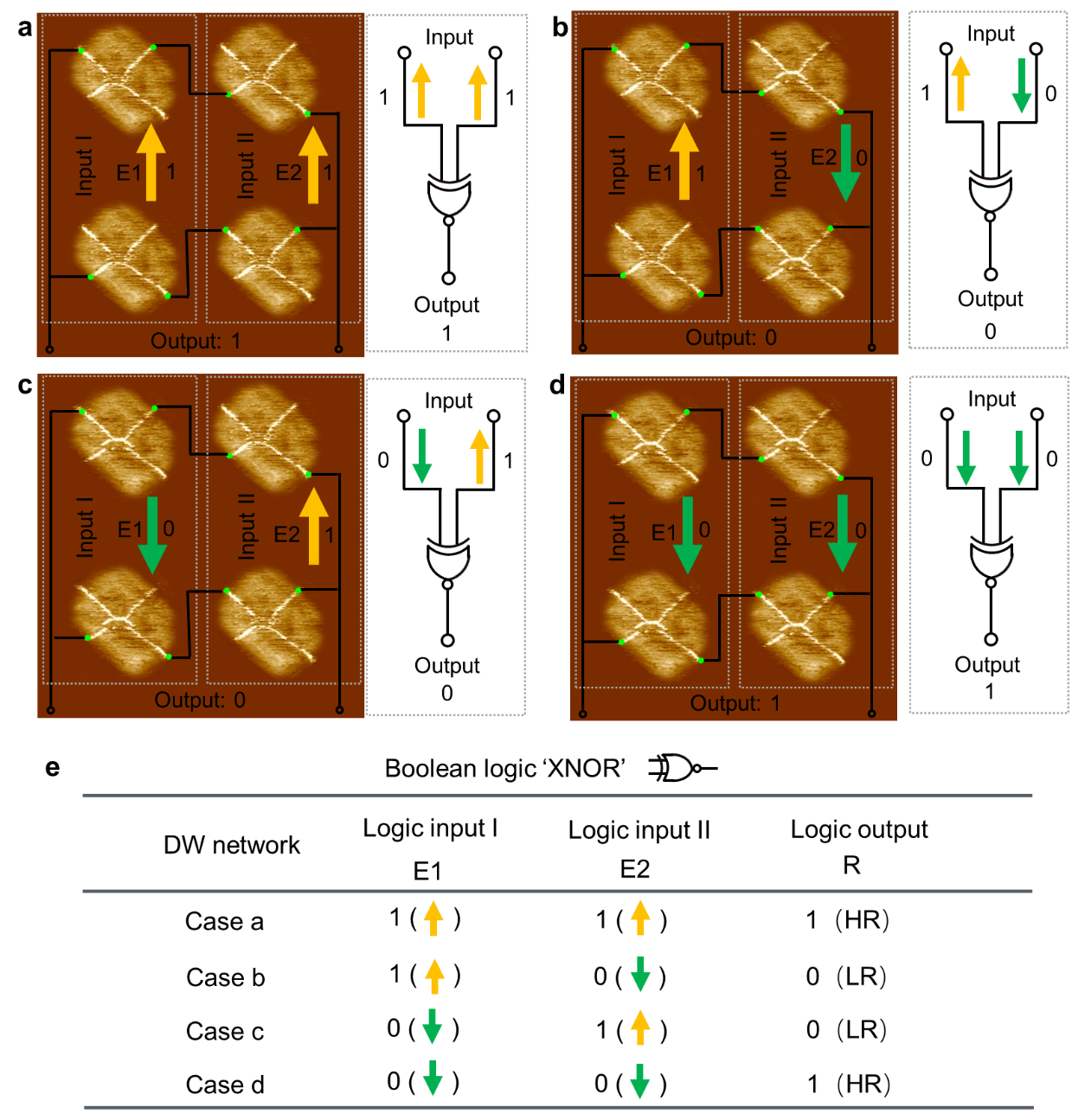


Supplementary Figure 19. Reconfigurable XNOR logic gate. **a**-**d**, Schematics of different logic operations for inputs of ‘11’, ‘10’, ‘01’, and ‘00’ when multiple nano-islands are joined in serious and parallel, respectively. **e**, Truth table for XNOR logic gate.


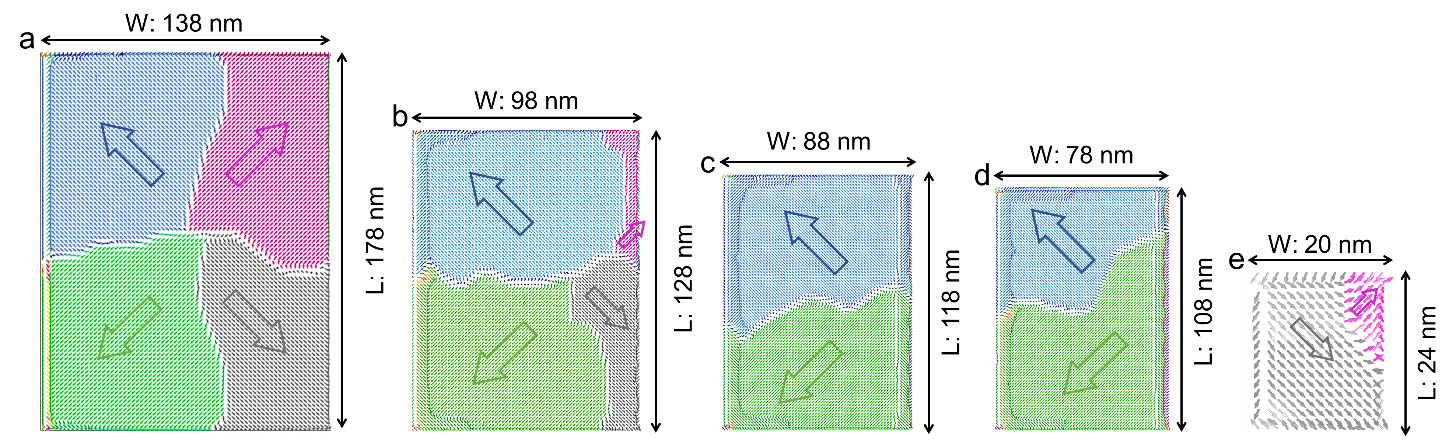


Supplementary Figure 20. Critical size for the formation of stable fourfold vertex-like quad-domain pattern in the rectangular BiFeO_3_ nano-islands. The four-fold quad-domain maintains when the nano-island size is decreased to 128 nm × 98 nm. The thickness of the nano-island keeps 7 nm.

Supplementary Table 2. List of coefficients used in the present work.

| Coefficients | Values |
| --- | --- |
| $\boldsymbol{\alpha}_{\boldsymbol{1}}$ | 4.64385×(T-1103)×10^5^ C^-2^m^2^N |
| $\boldsymbol{\alpha}_{\boldsymbol{11}}$ | 2.29047×10^8^ C^-4^m^6^N |
| $\boldsymbol{\alpha}_{\boldsymbol{12}}$ | 3.06361×10^8^ C^-4^m^6^N |
| $\boldsymbol{\alpha}_{\boldsymbol{111}}$ | 5.99186×10^9^ C^-6^m^10^N |
| $\boldsymbol{\alpha}_{\boldsymbol{112}}$ | -3.33980×10^8^ C^-6^m^10^N |
| $\boldsymbol{\alpha}_{\boldsymbol{123}}$ | -1.77754×10^9^ C^-6^m^10^N |
| $\boldsymbol{Q}_{\boldsymbol{11}}$ | 3.2×10^-2^ C^-2^m^4^ |
| $\boldsymbol{Q}_{\boldsymbol{12}}$ | -1.6×10^-2^ C^-2^m^4^ |
| $\boldsymbol{Q}_{\boldsymbol{44}}$ | 2.0×10^-2^ C^-2^m^4^ |
| $\boldsymbol{C}_{\boldsymbol{11}}$ | 302 GPa |
| $\boldsymbol{C}_{\boldsymbol{12}}$ | 162 GPa |
| $\boldsymbol{C}_{\boldsymbol{44}}$ | 68 GPa |

Supplementary References:

[1] A. Crassous, T. Sluka, A.K. Tagantsev, and N. Setter, Polarization Charge as a Reconfigurable Quasi-Dopant in Ferroelectric Thin Films, *Nat Nanotechnol*, **10**, 614-618 (2015).

[2] R.K. Vasudevan, Y. Matsumoto, X. Cheng, A. Imai, S. Maruyama, H.L. Xin, M.B. Okatan, S. Jesse, S.V. Kalinin, and V. Nagarajan, Deterministic Arbitrary Switching of Polarization in a Ferroelectric Thin Film, *Nat Commun*, **5**, 4971 (2014).

[3] C. Wang, X. Ke, J. Wang, R. Liang, Z. Luo, Y. Tian, D. Yi, Q. Zhang, J. Wang, X.F. Han, G. Van Tendeloo, L.Q. Chen, C.W. Nan, R. Ramesh, and J. Zhang, Ferroelastic Switching in a Layered-Perovskite Thin Film, *Nat Commun*, **7**, 10636 (2016).

[4] S.S.P. Parkin, M. Hayashi, and L. Thomas, Magnetic Domain-Wall Racetrack Memory, *Science*, **320**, 190-194 (2008).

[5] Z. Luo, A. Hrabec, T.P. Dao, G. Sala, S. Finizio, J. Feng, S. Mayr, J. Raabe, P. Gambardella, and L.J. Heyderman, Current-Driven Magnetic Domain-Wall Logic, *Nature*, **579**, 214-218 (2020).

[6] J. Li, B. Nagaraj, H. Liang, W. Cao, C.H. Lee, and R. Ramesh, Ultrafast Polarization Switching in Thin-Film Ferroelectrics, *Applied Physics Letters*, **84**, 1174-1176 (2004).

[7] C. Ma, Z. Luo, W. Huang, L. Zhao, Q. Chen, Y. Lin, X. Liu, Z. Chen, C. Liu, H. Sun, X. Jin, Y. Yin, and X. Li, Sub-Nanosecond Memristor Based on Ferroelectric Tunnel Junction, *Nat Commun*, **11**, 1439 (2020).

[8] S. Manipatruni, D.E. Nikonov, C.-C. Lin, T.A. Gosavi, H. Liu, B. Prasad, Y.-L. Huang, E. Bonturim, R. Ramesh, and I.A. Young, Scalable Energy-Efficient Magnetoelectric Spin–Orbit Logic, *Nature*, **565**, 35-42 (2018).

[9] R. Khosla and S.K. Sharma, Integration of Ferroelectric Materials: An Ultimate Solution for Next-Generation Computing and Storage Devices, *ACS Applied Electronic Materials*, **3**, 2862-2897 (2021).

[10] J.-M. Hu, Z. Li, L.-Q. Chen, and C.-W. Nan, High-Density Magnetoresistive Random Access Memory Operating at Ultralow Voltage at Room Temperature, *Nature Communications*, **2**, 553 (2011).
