# Supplementary material for: Ferroelectric domain-wall logic units
Source: Nat Commun. 2022 Jun 6;13:3255. doi: 10.1038/s41467-022-30983-4 (PMC9170692; doi:10.1038/s41467-022-30983-4)
Supplement: Supplementary file 2 — Description of Additional Supplementary Files [file 41467_2022_30983_MOESM2_ESM.pdf]

Description of Additional Supplementary Files

Supplementary Movie 1: Disruption of one CDW by $[\bar{1}\bar{1}0]$-oriented electric field.

Supplementary Movie 2: Connection of the disrupted CDW by [110]-oriented electric field.
